# Factors associated with SARS‐CoV‐2 infection in patients attending an acute hospital ambulatory assessment unit

**DOI:** 10.1002/jmv.26966

**Published:** 2021-04-06

**Authors:** Geoffrey Ronan, Lakshman Kumar, Mary Davey, Catriona O′Leary, Sarah McAleer, Jenny Lynch, Ros Lavery, John Campion, Joseph Ryan, P. J. O'Donoghue, Aine Daly, Jayne Shanahan, Sean Costelloe, Corinna Sadlier, Ciara McGlade, Sean Manning, Jennifer Carroll, Siun O'Flynn, Patrick Barry, Julio Chevarria

**Affiliations:** ^1^ Department of Radiology Cork University Hospital Cork Ireland; ^2^ University College Cork Cork Ireland

**Keywords:** coronavirus, epidemiology, pandemics, SARS coronavirus, virus classification

## Abstract

To describe the factors associated with severe acute respiratory syndrome coronavirus 2 (SARS‐CoV‐2) infection in mild‐to‐moderate patients attending for assessment. This observational study was conducted in a Model 4 tertiary referral center in Ireland. All patients referred for SARS‐CoV‐2 assessment over a 4‐week period were included. Patient demographics, presenting symptoms, comorbidities, medications, and outcomes (including length of stay, discharge, and mortality) were collected. Two hundred and seventy‐nine patients were assessed. These patients were predominantly female (62%) with a median age of 50 years (*SD* 16.9). Nineteen (6.8%) patients had SARS‐CoV‐2 detected. Dysgeusia was associated with a 16‐fold increased prediction of SARS‐CoV‐2 positivity (*p* = .001; OR, 16.8; 95% CI, 3.82–73.84). Thirteen patients with SARS‐COV‐2 detected (68.4%) were admitted, in contrast with 38.1% (99/260) of patients with SARS‐CoV‐2 non‐detectable or not tested (*p* = .001). Female patients were more likely to be hospitalized (*p* = .01) as were current and ex‐smokers (*p* = .05). We describe olfactory disturbance and fever as the main presenting features in SARS‐CoV‐2 infection. These patients are more likely to be hospitalized with increased length of stay; however, they make up a minority of the patients assessed. “Non‐detectable” patients remain likely to require prolonged hospitalization. Knowledge of predictors of hospitalization in a “non‐detectable” cohort will aid future planning and discussion of patient assessment in a SARS‐CoV‐2 era.

## Highlights

Mild‐to‐moderate patients with SARs‐CoV‐2 infection tend to present with characteristic olfactory disturbance.

From a medical assessment standpoint; patients with mild‐to‐moderate symptoms that are PCR negative remain likely to require hospitalization.

Mild‐to‐moderate patients with positive PCR testing are more likely to be admitted and require longer lengths of stay.

## 1 INTRODUCTION

The first cases of severe acute respiratory syndrome coronavirus 2 (SARS‐CoV‐2) infection, and coronavirus disease 2019 (COVID‐19), were reported in Europe between January 24 and February 21, 2020.^1^ In Ireland, the first documented cases were reported in late February. The first confirmed case at Cork University Hospital (CUH) was in a young male admitted with atypical pneumonia. Despite no epidemiological risks for COVID‐19, he was identified as positive by real‐time quantitative reverse transcription‐polymerase chain reaction (qRT‐PCR) on March 5th,^2^ just 2 months after the initial outbreak in Wuhan, China. As of July 13, 2020, a total of 2,581,512 cases have been reported within Europe with 196,773 deaths and 1,517,074 patients recovering.^3^ The cumulative incidence of COVID‐19 in Cork at the end of this study was 207.4 cases per 100,000.^4^

Limited data exists defining the presenting characteristics and outcomes of patients referred for assessment of potential COVID‐19 in Northern Europe. One observational study of European patients from 18 centers has suggested a highly variable presentation relative to age and sex. Olfactory dysfunction was important in mild‐to‐moderate patients.^5^ A retrospective cohort study of hospitalized patients testing positive for SARS‐CoV‐2 in China described a predominantly male cohort with a median age of 56 years.^6^ Mortality was 28% and 26% required intensive care unit admission. A summary of over 70,000 cases of SARS‐CoV‐2 in China highlighted that 81% of affected patients had mild‐to‐moderate disease.^7^ However, there are significant differences between the comorbidities of Northern European and Chinese populations.^8^

Currently, there are no studies characterizing the presenting symptoms and main risk factors for hospitalization in ambulatory SAR‐CoV‐2 positive patients presenting to acute medical assessment units. This study aimed to describe the population of patients presenting to a Model 4 tertiary referral center for SARS‐CoV‐2 assessment during the first wave of the pandemic and the factors affecting the risk of hospitalization in both SARS‐CoV‐2 detectable and non‐detectable cohorts.

The study describes the presenting features of patients with mild‐to‐moderate symptoms who met clinical criteria for suspected SARS‐CoV‐2, predictive symptoms, and factors affecting hospitalization. We expect that our results will inform the global community and health authorities about these differences and facilitate in the organization of their activity and management of resources.

## 2 METHODS

This observational study was conducted in CUH, a Model 4 tertiary referral center and university teaching hospital serving a population of over 1.1 million people in the South‐West of Ireland.

All consecutive patients referred to the SARS‐CoV‐2 assessment unit over a 4‐week period (March 30, 2020–April 26, 2020) were included. The unit was run by the acute internal medical service. Patients were isolated in separate rooms from presentation and provided with facemasks as per health authority recommendations.^9^

All healthcare professionals interacting with these patients wore full personal protective equipment (PPE). Staff was trained in donning and doffing PPE and Hazmat utilization. Patient imaging was performed in a designated area in the unit.

Pediatric and obstetric patients were excluded. Patients requiring intensive care and those deemed unstable were retained in the emergency department (ED). As a result, we assessed mild‐to‐moderate ambulatory patients.

**Figure 1:**
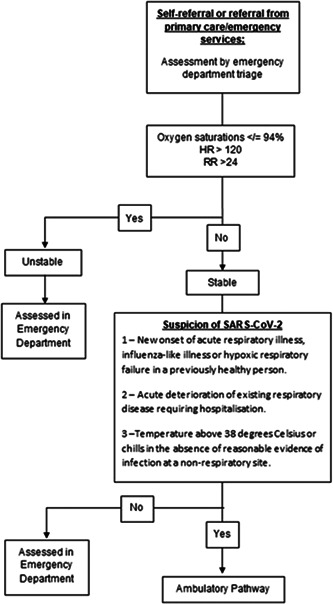
COVID‐19 assessment pathway heart rate (HR) and respiratory rate (RR). COVID‐19, coronavirus disease 2019; SARS‐CoV‐2, severe acute respiratory syndrome coronavirus 2

Patients seen had either been assessed in the ED by a triage service and deemed to meet assessment criteria (Figure 1) or were accepted directly by the assessment team based on a suspicion of SARS‐CoV‐2 in the setting of a potential need for hospitalization. Clinical outcomes were followed for 70 days.

For primary care and ambulance service; patients with suspected SARS‐CoV‐2 were referred for assessment where one of the following three clinical criteria was met: acute onset of at least one of cough, fever, shortness of breath AND no other etiology that explains the clinical presentation; the patient had any acute respiratory illness and had been in close contact with a confirmed or probable SARS‐CoV‐2 case in the 14 days before symptom onset; or sudden onset of anosmia, ageusia, or dysgeusia. A close contact as per international guidance was defined as <2 m of face‐to‐face contact, at <2 m distance, for >15 min. A probable case was defined as a suspected case for whom SARS‐CoV‐2 polymerase chain reaction (PCR) was negative but a clinical suspicion remained.

At entry to the hospital, patients were segregated into parallel SARS‐CoV‐2 and non‐SARS‐CoV‐2 streams. Criteria for entry into the COVID‐19 patient stream were defined as one of the following three. 1—New onset of acute respiratory illness, influenza‐like illness or hypoxic respiratory failure in a previously healthy person. 2—Acute deterioration of existing respiratory disease requiring hospitalization. 3—Temperature above 38°C or chills in the absence of reasonable evidence of infection at a non‐respiratory site.

A “stable patient” was defined based on three parameters: respiratory rate (RR), heart rate (HR), and oxygen saturations. Those with a RR > 24, HR > 120, and oxygen saturations ≤ 94% were retained in the ED and seen by the medical team under close monitoring. Those meeting the former criteria were transferred to the SARS‐CoV‐2 unit for assessment (Figure 1).

Data were collected from an admission proforma, patient notes, and an electronic database (Citrix iCM). Data collected included; patient demographics, smoking status, presenting symptoms, comorbidities, medications, vitals, clinical frailty score, laboratory tests, imaging, and outcomes (including length of stay, discharge, readmission, and mortality). Comorbidities were assessed using the Charlson Comorbidity Index.^10^

### 2.1 SARS‐CoV‐2 PCR testing

Specimens for SARS‐CoV‐2 testing were obtained as per the Centre for Disease Control and Prevention guidelines. Oropharyngeal and/or nasopharyngeal swabs were taken and placed in 3 ml transport media with real‐time RT‐PCR and RNA extraction thereafter.

Two DNA extraction kits were used based on availability; Roche Magnapure 24 system© and Indical bioscience Indimag 48 system©. Following this, one of three assays was used; Euroimmun SARS‐CoV‐2©, Serosep Respibio©, and Altona Realstar SARS‐CoV‐2 RT‐PCR Kit 1. Some data were analyzed using the Cethid Gene Xpert toward the end of the study period.

### 2.2 Statistical analysis

All statistical data were processed using IBM SPSS 19.0 statistical software. For the univariable analysis, the normality of the continuous variables was assessed using the Shapiro–Wilks test. The results are expressed as mean (*SD*) or median for continuous data, and as integers, frequencies, and percentages for categorical data.

We performed an exploratory analysis for categorical variables using the Fisher exact test or *χ* ^2^ test and continuous data with Student *t* test or *U* Mann–Whitney. The possible variables were selected among baseline variables and compared in a bivariable model, with patient main outcomes (risk for hospitalization) and to assess differences between SARS‐CoV‐2 detectable and non‐detectable patients. Results were considered statistically significant if the *p* value was <.05.

## 3 RESULTS

A total of 345 patients attended the COVID assessment unit. Sixty‐six patients were excluded from analysis. Four patients were incorrectly listed on the registry, 49 patients had no chart available, and 13 patients had five or more data missing. The total cohort studied post exclusion was 279.

These patients were mainly Caucasian (83.9%). There was a female predominance (62.2%) with a median age of 50 (*SD* 16.9). The majority of patients were never smokers (*n* = 177; 63%) with ex‐smokers and active smokers accounting for 22% (*n* = 63) and 14% (*n* = 39), respectively. Approximately one‐third of patients seen were being treated for an underlying respiratory condition (71 patients; 27%) (Table 1).

**Table 1:** Baseline characteristics

Characteristic	*n* = 279
Age—year mean (*SD*)	50 ± 16.9
Female sex, *n* (%)	173 (62.2%)
Race, *n* (%)	
Caucasian	234 (83.9%)
Non‐Caucasian	8 (2.9%)
Non‐specified	37 (13.2%)
Smoker, *n* (%)	
Current	39 (14.0%)
Ex	63 (22.6%)
Never	177 (63.4%)
Hypertension, *n* (%)	57 (22.3%)
Ischemic heart disease, *n* (%)	21 (8.3%)
Cardiac failure, *n* (%)	12 (4.8%)
Diabetes mellitus, *n* (%)	18 (7.1%)
Charlson Index, mean (*SD*)	1.49 (1.9)

**Figure 2:**
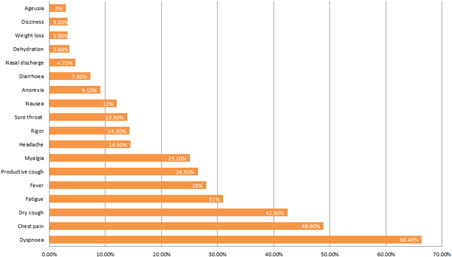
Presenting symptoms of all patients assessed

The most common presenting symptom was cough (69%) with 42.5% of patients reporting a dry cough and 26.5% a productive cough. Dyspnea was the second most common symptom affecting 66.4% of patients. Almost half of all patients attending reported chest pain (48.9%) and just under a third reported fatigue (31%). The full list of presenting symptoms is demonstrated in Figure 2.

Of the 279 patients seen, 186 were discharged (67%). One patient was discharged against medical advice and one was transferred to another hospital due to lack of bed space. The remaining 91 patients were admitted (33%). Two‐thirds of those admitted were isolated with suspected SARS‐CoV‐2 infection (*n* = 55; 67%).

The average length of stay of all patients admitted was 1.66 days (*SD* 3.93). For those discharged, the average time spent in the assessment unit was 5 h and 20 min (*SD* 2 h 19 min).

The average length of stay for SARS‐CoV‐2 positive patients admitted was 6.08 days (*SD* 6.5).

The most common diagnosis was lower respiratory tract infection (99 patients; 41.5%) with upper respiratory tract infection and musculoskeletal chest pain representing 10.1% and 6.7% of diagnoses, respectively.

**Figure 3:**
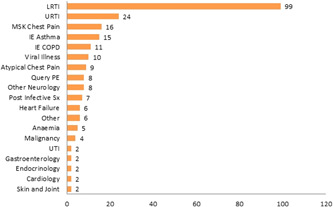
Diagnosis of patients assessed lower respiratory tract infection (LRTI), upper respiratory tract infection (URTI), musculoskeletal (MSK), infective exacerbation (IE), pulmonary embolism (PE), symptoms (Sx), and urinary tract infection (UTI)

A non‐respiratory diagnosis was made in approximately one‐third of patients (*n* = 74, 31.1%) (Figure 3).

**Figure 4:**
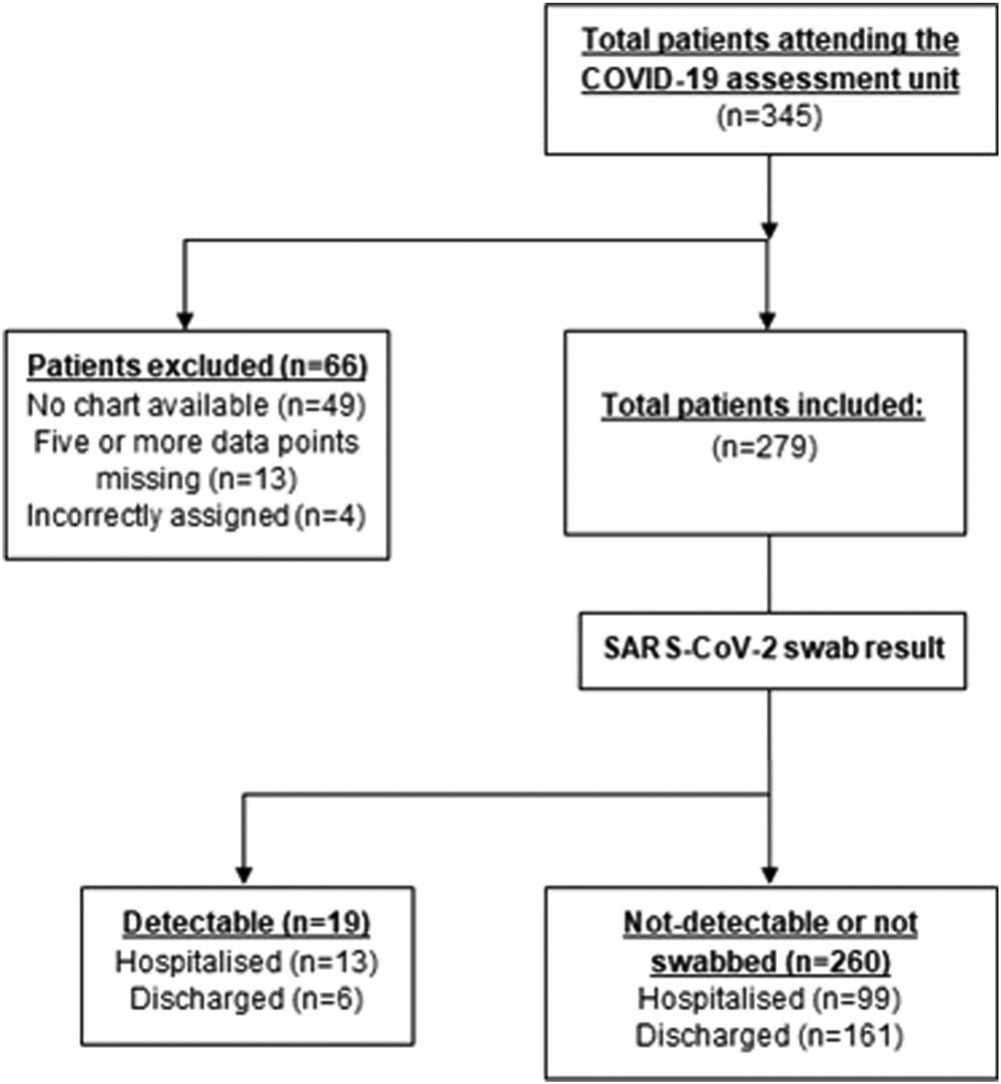
Hospitalization among SARS‐CoV‐2 detectable, not‐detectable, and patients not tested. COVID‐19, coronavirus disease 2019; SARS‐CoV‐2, severe acute respiratory syndrome coronavirus 2

One hundred sixty‐six patients (60%) had a combined oropharyngeal/nasopharyngeal swab for SARS‐CoV‐2, 76 patients did not meet local criteria for swabbing and the remaining 35 patients were swabbed in the community. Of the 166 patients swabbed, 19 were positive for SARS‐CoV‐2. A higher proportion, 68.4% (13/19), of patients with SARS‐CoV‐2 detectable by qRT‐PCR were hospitalized compared with 38.1% (99/260) of patients with SARS‐CoV‐2 not detectable by qRT‐PCR or not tested (*p* = .001).

There were a number of significant factors affecting hospitalization. Female patients were more likely to be hospitalized (*p* = .01) as were current and ex‐smokers (*p* = .05). Those with pre‐existing ischemic heart disease, cardiac failure, and hypertension were significantly more likely to be hospitalized (*p* = .04, *p* = .003, *p* = .03). This is evidenced concurrently by a significant association between the Charlson Comorbidity Index and hospitalization (*p* = .001). There was a significant association between hospitalization and presentation with; chest pain (*p* = .001) and anorexia (*p* = .04) (Table 2).

**Table 2:** Factors associated with hospitalization of patients assessed on the COVID‐19 assessment pathway

Characteristic	*p* value	OR (95% CI)
Age	.001	1.03 (1.02–1.05)
Female	.01	2.0 (1.2–3.4)
Current smoker	.05	1.4 (1.0–1.8)
Ex‐smoker	.05	1.4 (1.0–1.8)
Comorbidities		
Cardiac failure	.003	10.1 (2.2–47.3)
Hypertension	.03	1.9 (1.1–3.5)
Ischemic Heart Disease	.04	2.7 (1.1–6.6)
SARS‐CoV‐2 positive	.001	0.7 (0.7–0.8)
Presenting symptom		
Chest pain	.001	0.3 (0.2–0.4)
Anorexia	.04	2.4 (1.0–5.5)
Dehydration	.08	3.2 (0.9–11.5)
Dry cough	.09	0.6 (0.4–1.1)
Rigors	.09	1.8 (0.9–3.5)
Early Warning Score	.02	1.2 (1.0–1.4)
Charlson Index	.001	1.5 (1.3–1.7)

Abbreviations: CI, confidence interval; COVID‐19, coronavirus disease 2019; OR, odds ratio; SARS‐CoV‐2, severe acute respiratory syndrome coronavirus 2.

### 3.1 Factors associated with SARS‐CoV‐2 positivity

A total of 19 (6.8%) patients had SARS‐CoV‐2 detectable by qRT‐PCR of oropharyngeal/nasopharyngeal swab.

Dysgeusia was associated with a 16‐fold increase in SARS‐CoV‐2 positivity (*p* = .001; OR, 16.8; 95% CI, 3.82–73.84) as was an increase in respiratory rate (*p* = .02; OR, 1.15; 95% CI, 1.02–1.30) (Table 3). This is in keeping with international studies.^6^, ^7^

**Table 3:** Factors associated with SARS‐CoV‐2 PCR positivity

Variable	SARS‐CoV‐2 not‐detectable	SARS‐CoV‐2 detectable	OR (95% CI)
Male, *n* (%)	91 (35.1)	14 (73.7)	5.2 (1.8–14.8)
Female, *n* (%)	168 (64.9)	5 (26.3)	
Fever, *n* (%)	69 (26.5)	10 (52.6)	3.1 (1.2–7.9)
Shiver, *n* (%)	34 (13.2)	6 (31.6)	3.0 (1.1–8.5)
Dry cough, *n* (%)	109 (42.2)	9 (47.4)	1.2 (0.5–3.1)
Productive cough, *n* (%)	69 (26.5)	4 (21.1)	0.7 (0.2–2.3)
Dyspnea, *n* (%)	174 (67.2)	11 (57.9)	0.7 (0.3–1.7)
Chest pain, *n* (%)	129 (50.2)	4 (21.1)	0.3 (0.1–0.8)
Tiredness, *n* (%)	76 (29.3)	11 (57.9)	3.3 (1.3–8.6)
Headache, *n* (%)	38 (14.6)	2 (10.5)	0.7 (0.2–3.1)
Aches, *n* (%)	63 (24.2)	8 (42.1)	2.3 (0.9–5.9)
Sore throat, *n* (%)	37 (14.3)	1 (5.3)	0.3 (0.01–2.6)
Rhinorrhoea, *n* (%)	13 (5)	0 (0)	
Nausea, *n* (%)	30 (11.5)	3 (15.8)	1.4 (0.4–5.2)
Diarrhea, *n* (%)	18 (6.9)	2 (10.5)	1.6 (0.3–7.4)
Dehydration, *n* (%)	7 (2.7)	3 (15.8)	6.8 (1.6–28.6)
Anorexia, *n* (%)	19 (7.3)	6 (31.6)	5.9 (2.0–17.1)
Weight loss, *n* (%)	9 (3.5)	0 (0)	
Dysgeusia, *n* (%)	4 (1.6)	4 (21.1)	16.8 (3.8–73.8)
Dizziness, *n* (%)	9 (3.5)	1 (5.3)	1.5 (0.2–12.8)
SBP (mmHg), mean (*SD*)	136.5 (20.9)	127.9 (19.1)	0.98 (0.9–1.01)
DBP (mmHg), mean (*SD*)	81.4 (13.2)	76.2 (13.7)	0.97 (0.94–1.01)
Heart rate (bpm), mean (*SD*)	84.2(16)	84.9 (17.6)	1.03 (0.97–1.03)
Respiratory rate, mean (*SD*)	18.7 (3.5)	20.7 (3.6)	1.15 (1.02–1.30)
Saturation O_2_%, mean (*SD*)	97.8 (2.3)	96.6 (2.6)	0.88 (0.76–1.01)
Temperature (°C), mean (*SD*)	36.4 (0.6)	36.6 (0.7)	1.8 (0.9–3.8)

Abbreviations: °C, degrees celsius; CI, confidence interval; DBP, diastolic blood pressure; O_2_, oxygen; OR, odds ratio; SBP, systolic blood pressure; SARS‐CoV‐2, severe acute respiratory syndrome coronavirus 2; *SD*, standard deviation.

Factors associated with SARS‐CoV‐2 PCR positivity are shown in Table 3.

### 3.2 Laboratory findings

Analysis of laboratory parameters for SARS‐CoV‐2 detectable and SARS‐CoV‐2 non‐detectable cohorts revealed hyponatremia, eosinopaenia, and raised C‐reactive protein (CRP) as predictive markers for SARS‐CoV‐2 positivity (Table 4).

**Table 4:** Laboratory findings in SARS‐CoV‐2 detectable and non‐detectable patients

	SARS‐CoV‐2 non‐detectable mean (*SD*)	SARS‐CoV‐2 detectable mean (*SD*)	*p*
CRP (mg/L)	17.2(39)	58 (55.4)	.007
Sodium (mmol/L)	138.4 (3.5)	136.3 (3.2)	.02
Eosinophils (×10^9^/L)	0.15 (0.16)	0.06 (0.07)	.02
Basophils (×10^9^/L)	0.05 (0.02)	0.03 (0.02)	.02
Lactate (mmol/L)	1.4 (0.7)	1.9 (1)	.04
LDH (U/L)	369.7 (139.1)	477.9 (185.8)	.04
Hemoglobin (g/dl)	13.4 (1.8)	14.3 (1.6)	.05
Albumin (U/L)	42.5 (4.7)	40.1 (4.5)	.06
AST (U/L)	30.1 (24.7)	49 (23.5)	.06
Hematocrit (%)	38.6 (4.5)	40.7 (3.7)	.08
RBC (×10^12^/L)	4.5 (0.5)	4.7 (0.5)	.11
MCHC (g/dl)	34.5 (1.3)	35.1 (1.1)	.14
Creatinine (µmol/L)	75.2 (31.1)	84.9(26)	.20
CK (U/L)	95.7 (97.4)	125.2 (86.1)	.25
Platelets (×10^9^/L)	273.9 (89.8)	248.7 (91.7)	.29
WBC (×10^9^/L)	8.3 (3.3)	13.4 (20.7)	.36
Lymphocytes (×10^9^/L)	1.8 (1)	6.8 (20.7)	.37
Glucose (mmol/L)	6.1 (2.7)	6.6 (2.3)	.47
MCH (pg)	30 (2.5)	30.4 (1.2)	.47
Total bilirubin (µmol/L)	11.4 (8.5)	12.4 (4.9)	.66
Potassium (mmol/L)	4.2 (0.5)	4.1 (0.4)	.75
Troponin (ng/L)	28.1 (81.8)	18 (16.8)	.73
Neutrophils (×10^9^/L)	5.6 (3)	5.9 (2.2)	.73
ALT (U/L)	30.3 (27.3)	32.6 (18.6)	.73
Monocytes (×10^9^/L)	0.6 (0.3)	0.6 (0.3)	.76
Urea (mmol/L)	5.6 (3.9)	5.6 (1.6)	.93
MCV (fl)	86.7 (5.8)	86.8 (3.5)	.93

Abbreviations: ALT, alanine aminotransferase; AST, aspartate aminotransferase; CK, creatinine kinase; CRP, C‐reactive protein; MCH, mean corpuscular hemoglobin; MCHC, mean corpuscular hemoglobin concentration; MCV, mean corpuscular volume; SARS‐CoV‐2, severe acute respiratory syndrome coronavirus 2; WBC, white blood cell.

CRP was significantly raised in SARS‐CoV‐2 detectable patients (mean = 58.0 mg/L (0–5 mg/L)) compared with non‐detectable (mean = 17.2 mg/L (0–5 mg/L)) (*p* = .007). There was a mild reduction in serum sodium in SARS‐CoV‐2 detectable patients (mean = 136.3 mmol/L (132–144 mmol/L)) compared with non‐detectable patients (mean = 138.4 mmol/L (132–144 mmol/L) (*p* = .02).

Lymphocyte counts in SARS‐CoV‐2 detectable patients tended to be higher (mean = 6.8 × 10.9/L (1.5–4.5 × 10^9^/L)) compared with non‐detectable patients (mean = 1.9 × 10.9/L (1.5–4.5 × 10^9^/L)) though this was not statistically significant (*p* = .37). Serum eosinophils were significantly reduced in patients with SARS‐CoV‐2 detected (mean = 0.06 × 10^9^/L (0.04–0.4 × 10^9^/L)) compared with non‐detectable patients (mean = 0.15 × 10^9^/L (0.04–0.4 × 10^9^/L)) (*p* = .02). Interestingly eosinopaenia alone has been described as having high sensitivity and specificity for disease positivity.^11^, ^12^

### 3.3 Mortality

All‐cause mortality was 0.3% with a single‐case fatality during the period of study and follow‐up specified.

## 4 DISCUSSION

We report the clinical presentation and laboratory characteristics of 279 patients assessed on the COVID‐19 assessment pathway in a tertiary referral center in Ireland. To date, to the best of our knowledge, this is the first study reporting epidemiological and clinical features of European mild‐to‐moderate COVID‐19 disease with a direct comparison of patients who were SARS‐CoV‐2 not detectable at the time of presentation.

Our data suggest high presentation rates and hospitalization through a SARS‐CoV‐2 ambulatory assessment unit during the pandemic. This comes at a time of greatly reduced ED attendances and activity both nationally and internationally.^13^, ^14^ These studies highlight a concern that patients are foregoing necessary healthcare due to a fear of contracting SARS‐CoV‐2. They also describe a concurrent reduction in primary care referrals.^13^ No data currently exists for the long‐term effects of these trends on delayed diagnoses.

We describe a predominantly female cohort with a median age of 50, mild‐to‐moderate symptoms, and a low level of comorbidities. Among these patients, the most significant factors predicting hospitalization were SARS‐CoV‐2 status, pre‐existing cardiac disease, smoking status, chest pain, and female sex. Over two‐thirds of these patients were isolated with a suspicion of SARS‐CoV‐2 infection increasing the demand for isolation bed space.

Sparse data currently exists describing the presenting features and factors influencing hospitalization in patients with mild‐to‐moderate COVID‐19 disease. Lechien et al.^5^ describe a varying epidemiological and age‐related presentation among patients with mild‐to‐moderate COVID‐19 in Europe. Despite this variance, olfactory disturbance remains almost uniform among all groups.

Comparing SARS‐CoV‐2 detectable and not‐detectable patients, we report increased hospitalization rates and length of stay in those testing positive. In these patients, we describe characteristic symptoms of dysgeusia, fever, chest pain, and anorexia in a predominantly male population.

Though functioning as a SARS‐CoV‐2 ambulatory assessment unit, one‐quarter of all attendances to our department resulted in a non‐respiratory diagnosis. Furthermore, just 6.8% of those assessed tested positive for SARS‐CoV‐2 on viral PCR.

Risk factors increasing hospitalization demonstrate a propensity toward both respiratory and non‐respiratory symptomatology and comorbidities. The isolation rate of 67% for suspected SARS‐CoV‐2 among hospitalized patients suggests that a large cohort is being admitted for assessment of alternative diagnoses.

Peer review has recommended the isolation, dedicated imaging, and assessment of patients by staff trained in the appropriate use of PPE.^15^ A specific SARS‐CoV‐2 ambulatory assessment unit and pathway as described in this paper allows for these recommendations to be carried out appropriately.

The main limitation of this study is our small sample size of SARS‐CoV‐2 positive patients. Similarly, a proportion of assessed patients with COVID‐19 disease may have been non‐detectable at the time of testing by qRT‐PCR. With a limited number of cases, it is difficult to draw definitive conclusions, however, the total number of patients assessed presenting with SARS‐CoV‐2 symptomatology remains high and the implications of the structured approach toward their assessment is important for future planning. Further examination of future cohorts will be needed to appropriately assess our findings.

Though retrospective, this was a planned study with a strict protocol and detailed assessment proforma.

The choice of sampling site for viral PCR, nasopharyngeal/oropharyngeal, is limited and could lead to false positive and negative results, though it does remain the international standard.

Ahead of a possible “second wave” we continue to develop our assessment protocol based on the population presenting and statistical analysis of prior patients described here. Approaching the winter months, we expect an influx of patients with concurrent illnesses such as influenza A/B requiring independent isolation. Multiplex viral PCR may help inform assessment and treatment algorithms in these scenarios. Equally, with approximately one in four patients presenting diagnosed with non‐respiratory issues, a dedicated pathway for assessment of non‐SARS‐CoV‐2 patients presenting as mimics remains invaluable.

## 5 CONCLUSION

In conclusion, our multi‐dimensional study demonstrates that patients with mild‐to‐moderate SARS‐CoV‐2 infection present with characteristic olfactory disturbance and fever as overriding symptoms. These patients are more likely to be admitted and require a longer length of stay; however, they make up a minority of the patients assessed. Furthermore, those that do present for assessment and are not‐detectable for SARS‐CoV‐2 remain likely to require hospitalization and further diagnostics.

Knowledge of predictors of hospitalization, increased length of stay, and the necessity for complex diagnostics in a SARS‐CoV‐2 non‐detectable cohort will be helpful for future planning and discussion of on‐going assessment in a SARS‐CoV‐2 era.

## CONFLICT OF INTERESTS

The authors declare that there are no conflict of interests.

## AUTHOR CONTRIBUTIONS

All of the authors listed; Geoffrey Ronan, Lakshman Kumar, Mary Davey, C. O'Leary, Sarah McAleer, Jenny Lynch, Ros Lavery, John Campion, Joseph Ryan, P. J. O'Donoghue, Aine Daly, Jayne Shanahan, Sean Costelloe, Corinna Sadlier, Ciara McGlade, Sean Manning, Jennifer Carroll, S. O'Flynn, Patrick Barry, Julio Chevarria were involved in directing the care of the included patients, data collection, data analysis, or manuscript writing/editing.

## Data Availability

The data that support the findings of this study are available from the corresponding author upon reasonable request.
